# Supplementary material for: Convective dynamics in mantle of tidally-locked exoplanets
Source: Nat Commun. 2025 Jul 25;16:6846. doi: 10.1038/s41467-025-62026-z (PMC12297578; doi:10.1038/s41467-025-62026-z)
Supplement: Supplementary file 1 — Supplementary Information [file 41467_2025_62026_MOESM1_ESM.pdf]

Supplementary Information: Convective Dynamics in Mantle of Tidally-Locked Exoplanets

Daisuke Noto[1*], Takehiro Miyagoshi[2*], Tomomi Terada[3], Takatoshi Yanagisawa[3,4], Yuji Tasaka[4,3*]

[1]Department of Earth and Environmental Science, University of Pennsylvania, Philadelphia, PA 19104, USA.
[2]Research Institute for Value-Added-Information Generation, Japan Agency for Marine-Earth Science and Technology, Yokohama, 236-0001, Japan.
[3]Laboratory for Flow Control, Faculty of Engineering, Hokkaido University, Sapporo, 060-8628, Japan.
[4]Research Institute for Marine Geodynamics, Japan Agency for Marine-Earth Science and Technology, Yokosuka, 237-0061, Japan.

*Corresponding author(s). E-mail(s): dnoto@upenn.edu; miyagoshi@jamstec.go.jp; tasaka@eng.hokudai.ac.jp;

Parameters

We estimate the dimensionless parameters in the mantle of tidally-locked exoplanets. First, we characterize the length-to-height aspect ratios, which can be separately defined at the top (surface) and the bottom (core-mantle boundary) for the spherical domain. For the top, it is defined as

$$\mathcal{A}_{\mathrm{top}} = \frac{L_{\mathrm{top}}}{H} = \frac{\pi R}{H} = \frac{\pi}{\phi}, \tag{1}$$

where $L_{\mathrm{top}} = \pi R$ is the hemispheric perimeter at the top. Analogously, for the bottom, it is defined as

$$\mathcal{A}_{\mathrm{bot}} = \frac{L_{\mathrm{bot}}}{H} = \frac{\pi(R-H)}{H} = \pi\left(\frac{1}{\phi} - 1\right), \tag{2}$$

where $L_{\mathrm{bot}} = \pi(R-H)$ is the hemispheric perimeter at the bottom. To compare with the experimental results obtained in a rectangular domain in the main text, we can define the average length-to-height aspect ratio $\mathcal{A}$ as

$$\mathcal{A} = \frac{\mathcal{A}_{\mathrm{top}} + \mathcal{A}_{\mathrm{bot}}}{2} = \frac{\pi}{2}\left(\frac{2}{\phi} - 1\right). \tag{3}$$

For the case of Earth, where $H \approx 3000$ km and $R \approx 6500$ km ($\phi \approx 0.462$), the length-to-height aspect ratios are computed as $\mathcal{O}(1)$, $\mathcal{A}_{\mathrm{top}} \approx 6.81$, $\mathcal{A}_{\mathrm{bot}} \approx 3.67$, and $\mathcal{A} \approx 5.23$, and the experiments were performed in a rectangular vessel of $\mathcal{A} = 2$ ($\phi \approx 0.880$).

The Rayleigh number, the ratio of destabilizing buoyancy force to the viscous and thermal dissipation, is utilized as one of the essential parameters. For Rayleigh–Bénard convection (RBC) [1] and horizontal convection (HC) [2], it is often defined separately—the vertical Rayleigh number Ra_z and the horizontal Rayleigh number Ra_x—as

$$\mathrm{Ra}_z = \frac{g\,\alpha\,\Delta T\,H^3}{\kappa\,\nu} \quad \text{and} \quad \mathrm{Ra}_x = \frac{g\,\alpha\,\Delta\theta\,L^3}{\kappa\,\nu}, \tag{4}$$

where g, α, κ, and ν are the gravitational acceleration, the thermal expansion coefficient, the thermal diffusivity, and the kinematic viscosity. ΔT and $\Delta\theta$ are the temperature differences in the vertical and horizontal directions. However, the thermo-physical material properties of the mantle of tidally-locked exoplanets are largely unknown. Here, we utilize the following characteristic values from the mantle layers of Earth [3–5]:

- Gravitational acceleration $g = 10$ [m/s^2]
- Density $\rho = 4 \times 10^3$ [kg/m^3]
- Thermal expansion coefficient $\alpha = 1 \times 10^{-5}$ [1/K]
- Thermal diffusivity $\kappa = 1 \times 10^{-6}$ [m^2/s]
- Dynamics viscosity (lithosphere, top) $\mu_{\text{top}} = 1 \times 10^{24}$ [Pa $\cdot$ s]
- Dynamics viscosity (asthenosphere, bottom) $\mu_{\text{bot}} = 1 \times 10^{20}$ [Pa $\cdot$ s]
- Kinematic viscosity (lithosphere, top) $\nu_{\text{top}} = 2.5 \times 10^{20}$ [m^2/s]
- Kinematic viscosity (asthenosphere, bottom) $\nu_{\text{bot}} = 2.5 \times 10^{16}$ [m^2/s]

As listed above, the viscosity of mantle layers differs substantially between the top (lithosphere) and the bottom (asthenosphere). The Rayleigh numbers are thus defined for the top and the bottom as

$$\mathrm{Ra}_{z,\text{top}} = \frac{g\,\alpha\,\Delta T\,H^3}{\kappa\,\nu_{\text{top}}} \quad \text{and} \quad \mathrm{Ra}_{z,\text{bot}} = \frac{g\,\alpha\,\Delta T\,H^3}{\kappa\,\nu_{\text{bot}}}. \tag{5}$$

for the vertical Rayleigh number, and

$$\mathrm{Ra}_{x,\text{top}} = \frac{g\,\alpha\,\Delta\theta\,L_{\text{top}}^3}{\kappa\,\nu_{\text{top}}} = \mathcal{A}_{\text{top}}^3\,\Theta\,\mathrm{Ra}_{z,\text{top}} \quad \text{and} \quad \mathrm{Ra}_{x,\text{bot}} = \frac{g\,\alpha\,\Delta\theta\,L_{\text{bot}}^3}{\kappa\,\nu_{\text{bot}}} = \mathcal{A}_{\text{bot}}^3\,\Theta\,\mathrm{Ra}_{z,\text{bot}}, \tag{6}$$

for the horizontal Rayleigh number. Here, $\Theta = \Delta\theta/\Delta T$ is the degree of thermal nonuniformity. We can also define the hybrid Rayleigh number Ra as discussed in the main text, and it is defined as

$$\mathrm{Ra} = \frac{g\,\alpha\,(\Delta T + \Delta\theta)\,H^3}{\kappa\,\nu} = (1+\Theta)\,\mathrm{Ra}_z. \tag{7}$$

Accordingly, Ra for the top and the bottom is written as

$$\mathrm{Ra}_{\text{top}} = (1+\Theta)\,\mathrm{Ra}_{z,\text{top}} \quad \text{and} \quad \mathrm{Ra}_{\text{bot}} = (1+\Theta)\,\mathrm{Ra}_{z,\text{bot}}. \tag{8}$$

Because of the huge difference in viscosity at the top and the bottom, all Rayleigh numbers tend to be smaller at the top and larger at the bottom, setting the lower and upper limits of their ranges.

To constrain the length scale, H, we limit the super-Earth exoplanet's mass of interest up to 10 times that of Earth, i.e., the mass scale $\mathcal{M}$ ranges from 1 to 10. Providing that the material compositions of exoplanets are the same as those of Earth, the characteristic length of exoplanets is scaled by a factor $a = \sqrt[3]{\mathcal{M}}$, ranging from 1 to $\sqrt[3]{10}$ (≈ 2.15), i.e., R ranges from 6500 km to 14000 km and H ranges from 3000 km to 6463 km if ϕ is identical to that of Earth. We also fix the vertical temperature difference, $\Delta T = 3000$ K, the same as the characteristic ΔT of Earth's mantle. As an example, we consider $\Delta\theta = 1000$ K based on the recent observation [6, 7], yielding $\Theta = 1/3$. The estimation is shown in Fig. S1 in the Ra_z-Ra_x domain (Fig. S1a) and the Ra-Θ (Fig. S1b). Color represents the mass scale $\mathcal{M}$, and the hatched region corresponds to the parameter ranges of the present experiments. The range of Ra_z is $\mathcal{O}(10^4\text{-}10^9)$, whereas that of Ra_x is $\mathcal{O}(10^6\text{-}10^{10})$ for the exoplanets. These ranges widely overlap with those of laboratory experiments, upholding the discussion provided in the main text.

Convective regimes and hybrid Rayleigh number

We identify three convective regimes that build on time-dependency: steady, periodic, and unsteady regimes. The regimes can be distinguished by the temporal evolution of measured quantities such as Re, and differences emerge as distinctive differences in their power spectra. The experimental parameters we explored in the main text are plotted in the Ra-Θ domain (Fig. S2a). Three different symbols, circles, squares, and diamonds, correspond to different Pr, 10^2, 10^3, and 10^4, respectively. The vertical dashed line is an empirical border distinguishing steady and unsteady convective motions, $\mathrm{Ra} = 4.7 \times 10^6$. We also replot the Re evolution provided as Fig. 2d in the main text in Fig. S2b. The corresponding power spectral density (PSD), obtained for the Re profile in $4000 < t < 5000$, is shown in Fig. S2c. The periodic case shows a distinctive peak at a specific frequency $f \approx 0.018$.

We demonstrate the excellence of the hybrid Rayleigh number Ra as the primary control parameter by showing the collapse of the Péclet number Pe in Fig. 2e. Here, we show the comparison of different Rayleigh numbers in Fig. S3.

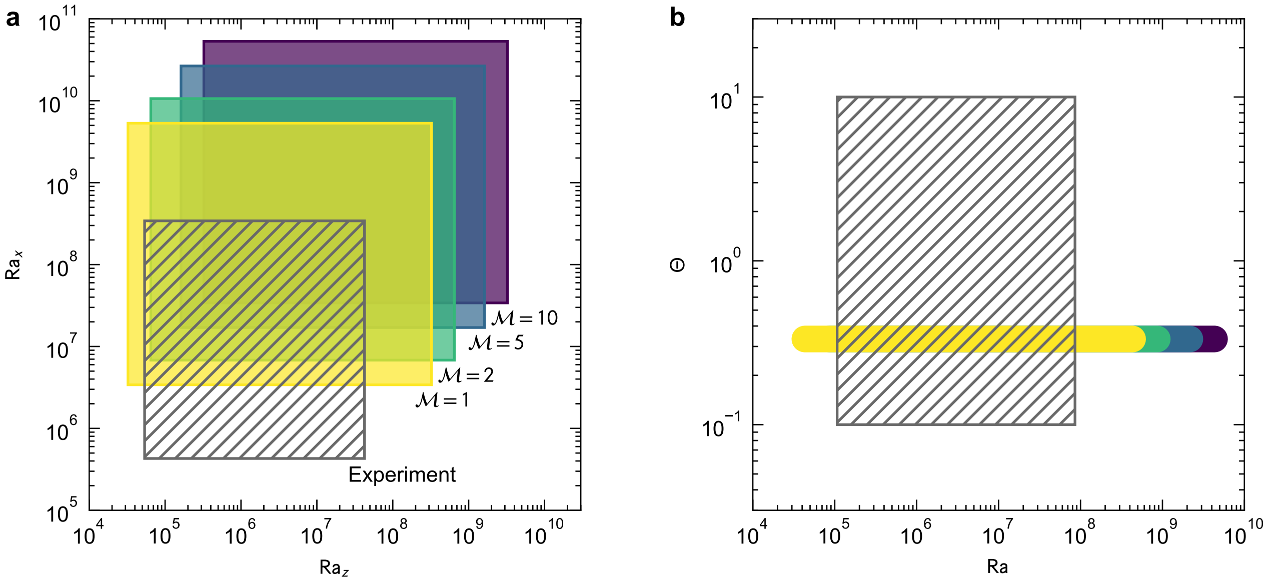

Fig. S1 Parameter space of mantle layers in tidally-locked exoplanets. a Ra_z v.s. Ra_x. **b** Ra v.s. Θ. Color represents different exoplanet masses, $\mathcal{M} = 1$, 2, 5, and 10, and the hatched region is the range covered by the experiments. For the estimation of parameter ranges of tidally-locked exoplanets, we fix $\phi = 0.462$ and $\Theta = 1/3$.

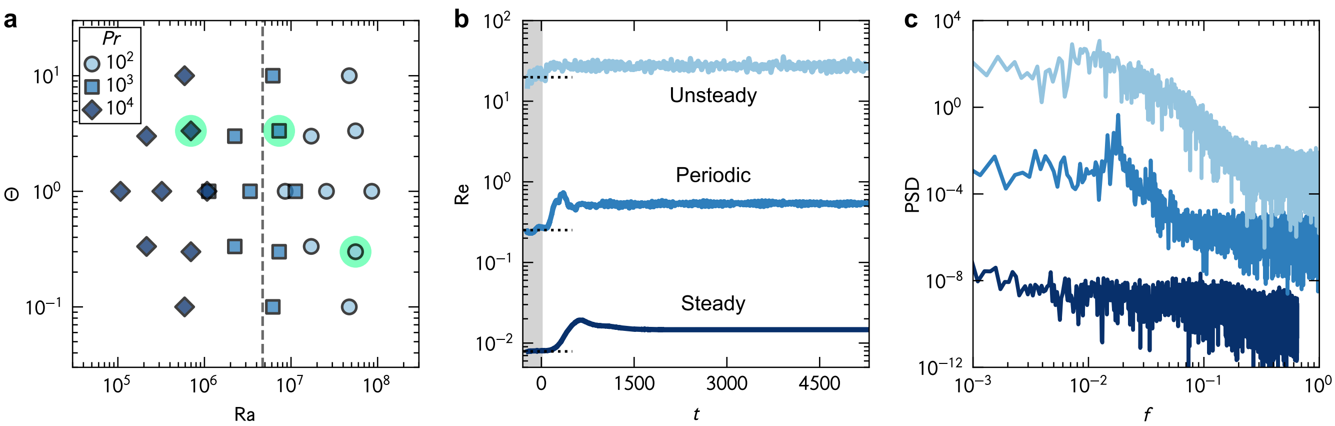

Fig. S2 Convective regimes. a Explored parameters mapped in the Ra-Θ domain. Circles, squares, and diamonds correspond to $\mathrm{Pr} = 10^2$, 10^3, and 10^4, respectively. The dashed line is an empirical threshold for distinguishing steady and unsteady convective motions, $\mathrm{Ra} = 4.7 \times 10^6$. **b** Temporal evolutions of the Reynolds number Re (replot of Fig. 2d in the main text). **c** Power spectra of the Re signals plotted in **b**.

While Ra_z and Ra_x, shown in Fig. S3a and b, exhibit scattered distributions due to the insufficient consideration of total available potential energy, Ra shows an excellent collapse, proving the propriety of Ra as the key parameter. However, further theoretical analysis and extensive parametric studies are needed to construct rigorous scaling laws for heat transport, i.e., Nu-Ra relationship shown in Fig. 3f and g.

References

[1] Ahlers, G., Grossmann, S. & Lohse, D. Heat transfer and large scale dynamics in turbulent Rayleigh–Bénard convection. *Rev. Mod. Phys.* **81**, 503 (2009).

[2] Hughes, G. O. & Griffiths, R. W. Horizontal convection. *Annu. Rev. Fluid Mech.* **40**, 185–208 (2008).

[3] Stein, C. A. & Stein, S. A model for the global variation in oceanic depth and heat flow with lithospheric age. *Nature* **359**, 123–129 (1992).

[4] Gordon, R. G. *Diffuse Oceanic Plate Boundaries: Strain Rates, Vertically Averaged Rheology, and Comparisons With Narrow Plate Boundaries and Stable Plate Interiors*, 143–159 (American Geophysical Union (AGU), 2000).

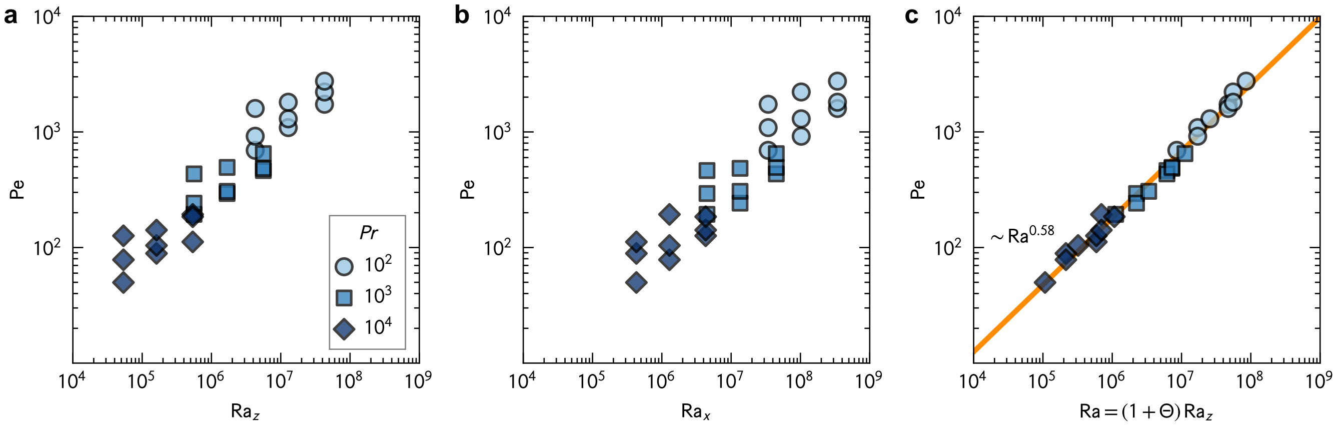

Fig. S3 Péclet number v.s. Rayleigh number. **a** Pe v.s. Ra_z, the vertical Rayleigh number. **b** Pe v.s. Ra_x, the horizontal Rayleigh number. **b** Pe v.s. Ra, the hybrid Rayleigh number. The solid line is the power-law fitting, $\mathrm{Pe} \sim \mathrm{Ra}^{0.58}$, which agrees well with the scaling $\mathrm{Pe} \sim \mathrm{Ra}_z^{2/3}$ obtained for RBC [8, 9].

[5] Whittington, A. G., Hofmeister, A. M. & Nabelek, P. I. Temperature-dependent thermal diffusivity of the Earth's crust and implications for magmatism. *Nature* **458**, 319–321 (2009).

[6] Kreidberg, L. *et al.* Absence of a thick atmosphere on the terrestrial exoplanet LHS 3844b. *Nature* **573**, 87–90 (2019).

[7] Lyu, X. *et al.* Super-Earth LHS3844b is tidally locked. *Astrophys. J.* **964**, 152 (2024).

[8] Grossmann, S. & Lohse, D. Thermal convection for large Prandtl numbers. *Phys. Rev. Lett.* **86**, 3316 (2001).

[9] Shishkina, O., Emran, M. S., Grossmann, S. & Lohse, D. Scaling relations in large-Prandtl-number natural thermal convection. *Phys. Rev. Fluids* **2**, 103502 (2017).
