# Supplementary material for: Convective dynamics in mantle of tidally-locked exoplanets
Source: Nat Commun. 2025 Jul 25;16:6846. doi: 10.1038/s41467-025-62026-z (PMC12297578; doi:10.1038/s41467-025-62026-z)
Supplement: Supplementary file 2 — Description of Additional Supplementary Files [file 41467_2025_62026_MOESM2_ESM.pdf]

Description of Additional Supplementary Files

File name: Supplementary Movie 1
Description: Convective dynamics in tidally-locked mantle for steady regime. Laboratory experiment (left) and its hemispheric projection (right).

File name: Supplementary Movie 2
Description: Convective dynamics in tidally-locked mantle for peridic regime. Laboratory experiment (left) and its hemispheric projection (right).

File name: Supplementary Movie 3
Description: Convective dynamics in tidally-locked mantle for unsteady regime. Laboratory experiment (left) and its hemispheric projection (right).
